# The effect of size, sex and running discipline on the shape of rectus abdominis muscle: A 3D geometric morphometric approach

**DOI:** 10.1371/journal.pone.0358842

**Published:** 2026-10-28

**Authors:** Marta Gómez-Recio, Benoit Beyer, Elena García-Vargas, Vladimír Sládek, Markus Bastir

**Affiliations:** 1 National Museum of Natural Sciences, Madrid, Spain; 2 Department of Biology, Faculty of Sciences, Universidad Autónoma de Madrid (UAM), Madrid, Spain; 3 Université Libre de Bruxelles, Laboratory of Anatomy Biomechanics and Organogenesis, Brussels, Belgium; 4 Department of Anthropology and Human Genetics, Faculty of Sciences, Univerzita Karlova, Prague, Czech Republic; University of Montenegro: Univerzitet Crne Gore, MONTENEGRO

## Abstract

The morphology of the rectus abdominis muscle is closely related to its biomechanical functions in trunk stabilization and breathing across different activities. However, the three-dimensional morphological characteristics of this muscle remain poorly documented in vivo. Therefore, the present observational cross-sectional study aims to evaluate how size, sex and running discipline influence the 3D shape of this muscle, hypothesising that there are allometric effects, sexual dimorphism effects and that the explosive demands of sprinting require a more specialised morphology than endurance running. This work models the in vivo shape of this muscle at rest in a sample of athletes specialised in endurance running or sprinting. The rectus abdominis muscles were measured directly from CT scan slices, and 3D geometrical models of the muscle were created from the triangulation of 3D coordinates. The shape of the muscle was studied through 3D geometric morphometric analyses. The study revealed the significant effect of sex and running discipline in the 3D shape of the rectus abdominis. The results highlight distinct morphological features in males and females and in sprinters and endurance runners. Males show a more triangularly shaped rectus abdominis (wider in its superior part) compared to females, who show a more rectangular muscle. Similarly, sprinters show thicker and more rectangular muscles, compared to endurance runners. These findings suggest that the functional anatomy of the rectus abdominis muscle could differ depending on sex and running discipline, pointing to potential differences in biomechanics and respiratory performance. This novel approach opens the door to further exploration of in vivo muscle morphology and its implications in physiology applied not only to sports science, but also in clinical settings.

## Introduction

The rectus abdominis is one of the five muscles that build up the anterior abdominal wall. It is a paired, vertically oriented muscle that originates from two tendinous attachments to the pelvis. One broad tendon attaches to the pubic crest, often extending to the pecten pubis, while the other attaches to the anterior surface of the pubic symphysis. Superiorly, the muscle inserts into the xiphoid process and the 5th to 7th costal cartilages, although the exact insertion points may vary slightly among individuals [1]. To date, several high resolution 3D atlases of human muscles have been created, including those of the anterior abdominal wall muscles [2].

Functionally, this muscle plays a key role not only in trunk flexion and posture, but also in breathing mechanics [3–5]. Like all other anterior abdominal wall respiratory muscles, it participates in the expiratory function as a pressure generator [3]. Specifically, the rectus abdominis muscle is activated in voluntary expiration from functional residual capacity [5] and during CO2 stimulated ventilation [6].

During exercise, muscles demand more oxygen and the physiology of breathing changes. For instance, the role of the diaphragm changes from pressure generator to flow generator, contracting faster, and the abdominal muscles take the role of generating pressure, pushing the diaphragm superiorly towards the lungs during expiration [3]. After expiration, the reduced lung volume creates an elastic pressure which is stored as potential elastic energy which will be used to help start the inspiratory movement [5–7]. However, athletes push this further, reaching the mechanical limits of their respiratory system, producing the maximum inspiratory and expiratory pressures that the airways can undergo [8].

The rectus abdominis, as well as the rest of the abdominal respiratory muscles plays a passive role during inspiration. Therefore, although its main role in the respiratory function during exercise is accessory, its contribution to expiratory pressure generation during exercise should not be underestimated. Nonetheless, most literature regarding the rectus abdominis is limited by small sample size and studies are usually conducted in few male subjects. Considering this, findings from many studies might not be generalisable.

There is a lack of understanding of how size, sex or type of athletic training in running (sprinting or endurance running) may influence abdominal muscles morphology. This creates a significant knowledge gap which can now start to be covered through pioneering 3D modelling of in vivo muscles in broader samples. To first understand the in vivo morphological variation of this muscle in humans, the application of translational methodologies such as 3D geometric morphometrics could be key to understand morphological differences between subjects. This study aims to characterize the three-dimensional shape variation of the rectus abdominis muscle in a sample of endurance and sprint athletes, hypothesising that size, sex and running discipline significantly influence muscle morphology.

## Materials and methods

Thirty-six competitive runners (21 males, 15 females, ages from 18 to 45) were recruited for this observational cross-sectional study (Table 1). Based on self-reported training data obtained via a structured questionnaire, participants were classified by sex and running discipline, creating the following group combinations: female sprinter, female endurance, male sprinter and male endurance. Participants were excluded if they had a medical history of cardiovascular pathologies, lactate problems or if they presented a noticeable diastasis or any abnormalities related to the rectus abdominis muscle. All participants were informed of the study procedures and provided written informed consent prior to participation. The study was conducted under the initial approval of the ethics committee (IRB Univerzita Karlova 2022/12), with subsequent protocol expansions and renewals approved in 2023 and 2024 (IRB Univerzita Karlova 2024/03) to incorporate additional procedures [9]. Recruitment and data collection took place from November 16, 2023, to March 21, 2025, in accordance with the Declaration of Helsinki.

**Table 1:** Descriptives of the sample.

		Age (years)	Body height (cm)	Body weight (kg)
Female sprinter	N	8	8	8
	min	19	153	55
	max	32	170	65.5
	mean	24.13	163.81	59.71
	s.d.	4.58	6.64	4.00
Female endurance	N	7	7	7
	min	31	152	48.3
	max	43	165	56.5
	mean	37.57	159.29	51.66
	s.d.	4.76	5.94	2.97
Male sprinter	N	10	10	10
	min	18	168	61.5
	max	27	183	75.4
	mean	21.10	176.55	69.55
	s.d.	3.28	4.72	4.54
Male endurance	N	11	11	11
	min	20	171	55
	max	45	184	83.5
	mean	32.64	176.55	68.65
	s.d.	8.30	4.23	9.46

Given the exploratory nature of 3D in vivo geometric morphometric studies and the logistical constraints of CT-based data collection in athletic populations, no formal a priori power calculation was performed. Sample size was determined by feasibility of recruitment within the study period and is comparable to, or larger than, sample sizes used in prior in vivo morphometric studies (e.g., [10]). We acknowledge this as a limitation affecting statistical power (see Discussion).

Following basic anthropometric measurements (Table 1), participants underwent CT scanning in supine position at the Centro Nacional de Aceleradores (Sevilla, Spain). Biograph mCT systems were operated using standardized settings: 0.6 mm slice width, 0.5 mm reconstruction increment, 64x0.6mm collimation, pitch 0.8, 120 kVp, 160 mAs, and a rotation time of 0.5 s. During CT acquisition from apex to base, participants were coached to perform breath-holds at two lung volumes: TLC (deep inspiration) and FRC (end-tidal expiration). These acquisitions were conducted as part of a broader, multidisciplinary research project designed to comprehensively evaluate breathing function of upper body structures, including the thorax, cervical spine, lungs, and upper airways. However, to strictly adhere to bioethical principles regarding radiation safety and minimize unnecessary secondary exposure to the gonadal region in healthy participants, the acquisition window was intentionally terminated in several instances prior to reaching the pubic symphysis in the TLC manoeuvre. Consequently, only the FRC data were analysed to ensure full 3D reconstruction and measurement of the rectus abdominis muscle. High-resolution images (B30f) were reconstructed in DICOM format for subsequent 3D modelling.

The high-definition CT allowed enough resolution to detect the rectus abdominis muscle in the transversal slices, and the muscle could be 3D modelled. All subjects were measured using Amira 4.0 software (Thermo Fisher Scientific, Waltham, MA, USA) [11]. The shape registration process (Fig 1) consisted of creating a simplified template that collected the overall shape of the rectus abdominis.

**Fig 1:**
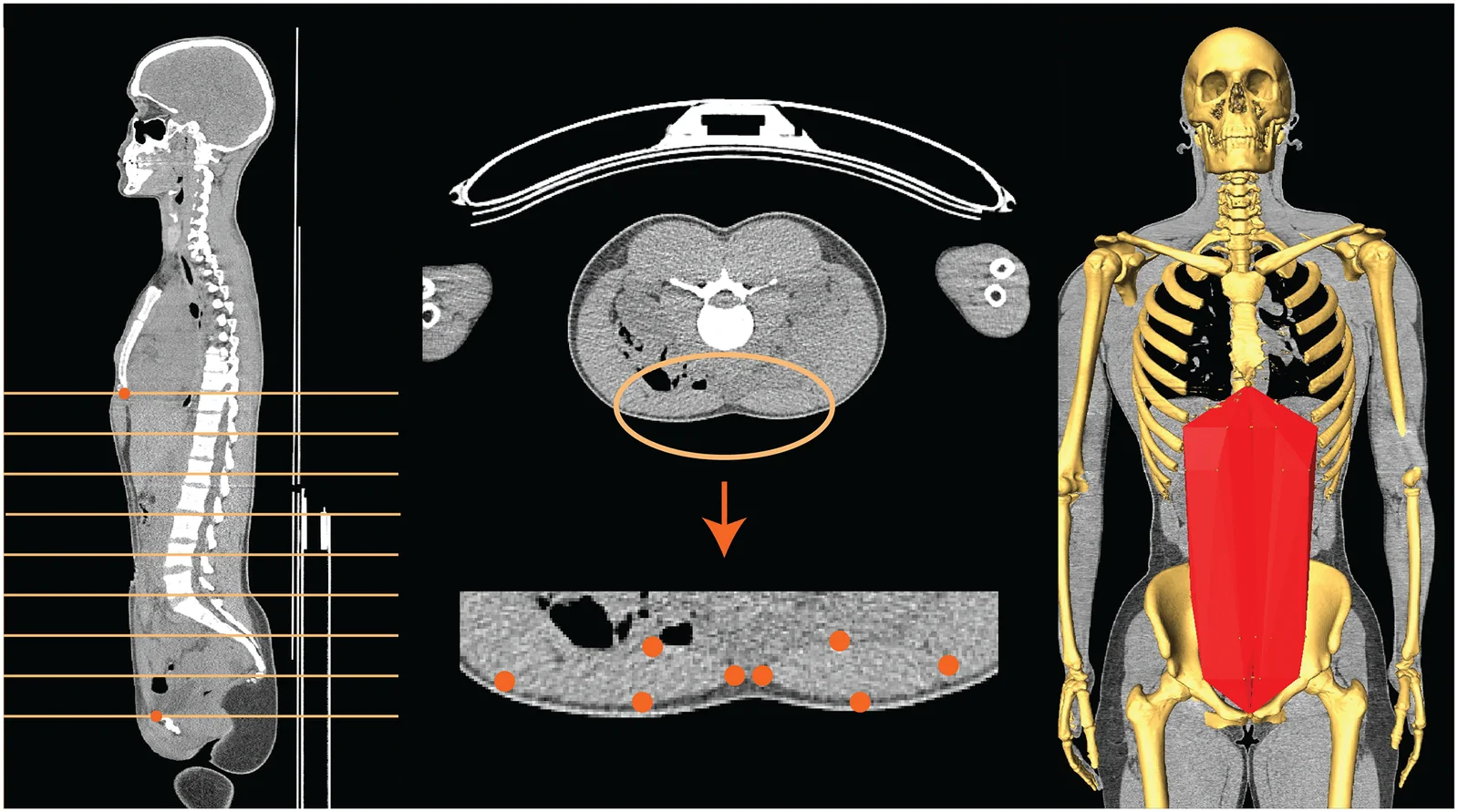
Shape registration of the rectus abdominis muscle in a subject of the sample. A. Equidistant division of the seven levels of the muscle that were measured. B. Eight landmarks taken at each of the equidistant levels, collecting the rectus abdominis shape. C. 3D convex hull model of the rectus abdominis muscle.

Shape was measured through 3D geometric morphometrics methods, using 3D cartesian coordinates called landmarks to collect information about the shape and size of the muscle [12]. Landmark placement followed a standardized protocol: the same trained observer placed all landmarks directly on the axial CT slices using Amira 4.0 software (Thermo Fisher Scientific, Waltham, MA, USA) [11], guided by the template in Fig 1B. We placed the first anatomical landmark sagittally, on the ending point of the xiphoid process. The next landmark was taken also sagittally, on the anterior superior pubic symphysis. Afterwards, 9 equidistant levels, perpendicular to the lateral view of the CT were defined. To establish the 9 equidistant levels, the total number of axial CT slices between the xiphoid process (Level 1) and the pubic symphysis (Level 9) was determined for each subject. This total slice distance was divided by 8 to obtain a constant slice interval. The 7 intermediate levels (Levels 2–8) between the first two anatomical landmarks were then located sequentially by subtracting this fixed interval from the xiphoid process slice number (larger than the pubic symphysis slice number). At each of these 7 internal levels, measurements were performed as shown in Fig 1B, dividing the rectus abdominis muscle into right and left sides and using 4 geometrical landmarks per level and side to best capture the shape of the muscle. This process resulted in a total registration of 58 landmarks per subject. Formal intra- and inter-observer reliability testing was not performed in this study.

The shape configurations of all individuals were subjected to Procrustes superimposition, also known as Generalized Procrustes Analysis (GPA) [13]. This procedure minimises the differences between landmarks by removing the effects of position, rotation and scale of the different configurations. This way we can define shape as the geometric properties of an object that remain invariant after GPA. GPA will allow the independent analysis of shape and size by calculation of centroid size (CS). This is a measure of size calculated as the square root of the sum of squared distances between each landmark and the geometrical mean of all landmarks (the centroid). The resulting Procrustes coordinates were analysed through PCA. This first approach to exploring the sample allowed us to understand the principal directions of shape variation, depicted through 3D shape deformations of the geometrical model of the rectus abdominis.

The statistical approach used to test our main hypotheses regarding the effects of size, sex and running discipline on muscle morphology was carried out through a Procrustes ANOVA model [14]. We tested a complete multivariate model, assessing the effects of size (to evaluate allometry), sex (to evaluate sexual dimorphism) and running discipline (to evaluate sport specialisation) on shape, including interaction between these factors. The model was subsequently simplified based on the absence of significance of interactions. The effect of size, sex and running discipline on shape was explained through linear and dummy regression models and 3D shape deformations representing the extremes of shape variation associated with each factor.

All geometric morphometric analyses, plots and 3D visualizations were made in R. Convex hull models of the rectus abdominis were smooth shaded and we applied an orthogonal view filter for graphic purposes in Artec Studio 16 professional software.

The manuscript was reported according to the STROBE (Strengthening the Reporting of Observational Studies in Epidemiology) guidelines (S2 Table).

## Results

The PCA (Fig 2) projects the different rectus abdominis shape configurations onto a common shape space defined by the principal components and shows the principal axes of shape variation. PC1 and PC2 explain 47.31% of total shape variation.

**Fig 2:**
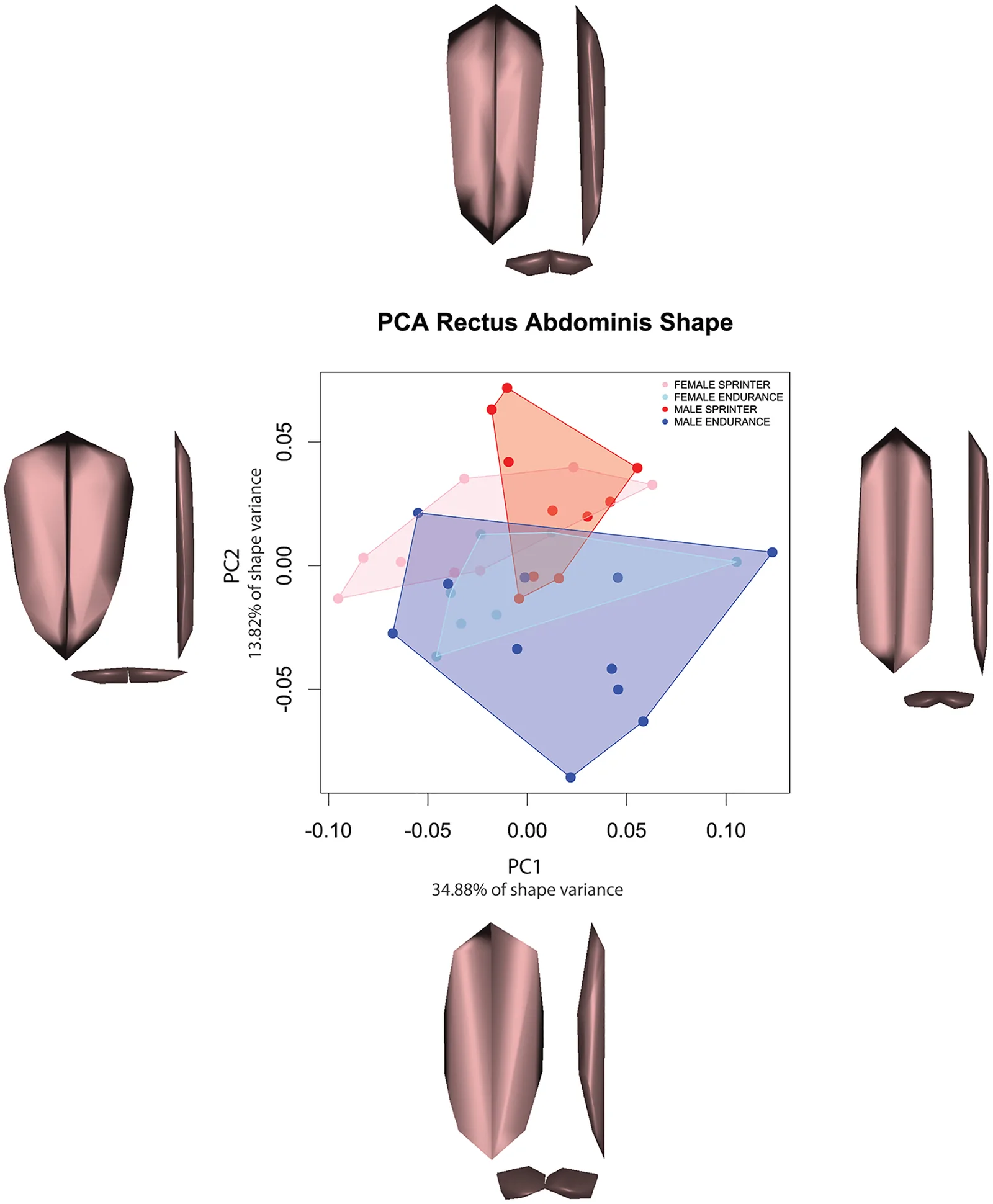
Principal component analysis exploring the main axes of shape variation of the rectus abdominis muscle.

PC1 primarily reflects variation in the superior region of the rectus abdominis muscle. Smaller values of PC1 indicate more rectangular rectus abdominis muscles, superiorly thicker, while larger values are associated to an inverted triangular shape of the muscle, superiorly thinner. We can see overlap between the endurance and sprinter groups in this first principal component, although there is a greater difference between males and females of the sprinter group. Larger PC2 values indicate a rectus abdominis that is wider and thicker superiorly, with a convex muscle shape. In contrast, smaller PC2 values correspond to a rectus abdominis that is wider and thicker in the middle portion, exhibiting a concave shape. Endurance runners tend to show lower PC2 values, while sprinters show larger values. Overall, males show greater variance than females.

The first Procrustes ANOVA approach revealed there was no interaction between centroid size, sex and running discipline (S1 Table). Therefore, the model was simplified to not consider interaction, as shown in Table 2. The analysis shows significant effects of centroid size, sex and running discipline on the rectus abdominis shape. However, as the R^2^ shows, these factors account only for a small proportion of shape variation (Table 2). The rectus abdominis muscle is shaped by its size, expressing the allometric pattern shown in Fig 3A. Smaller rectus abdominis muscles are superiorly wider and inferiorly thicker than larger ones. The effect of sex, although significant, is less noticeable in general terms, but is stronger in sprinters (Fig 3B). However, this stronger sexual dimorphism in sprinters was observed as a trend as there was no significant effect of the interaction between sex and running discipline (S1 Table). We observe that female rectus abdominis present a rectangular shape while male rectus abdominis are wider and thicker in its superior part, describing a component of shape variation similar to PC1. The effect of running discipline (Fig 3C) is similar to that of PC2, and shows that the rectus abdominis of endurance runners are wider and concave, while sprinters show narrower and more convex muscles.

**Table 2:** Procrustes ANOVA model exploring the effect of size (CS), sex and running discipline on shape (without interaction between factors).

	Df	SS	MS	R^2^	F	Z	p-value
CS	1	0.014	0.014	0.054	2.093	1.819	0.035*
Sex	1	0.016	0.016	0.062	2.433	2.003	0.023*
Running discipline	1	0.017	0.017	0.063	2.455	2.083	0.013*
Residuals	32	0.215	0.007	0.821			
Total	35	0.262					

Df, degrees of freedom; SS, sums of squares; MS, mean squares; R^2^, predicted variance; F, F-statistic; Z, effect size based on F distributions; p-value, significance level based on 1000 permutations. Type I (sequential) Sums of Squares were used to strictly control for allometric effects by accounting for centroid size (CS) prior to testing biological and discipline factors. Significance level*: p < 0.05.

**Fig 3:**
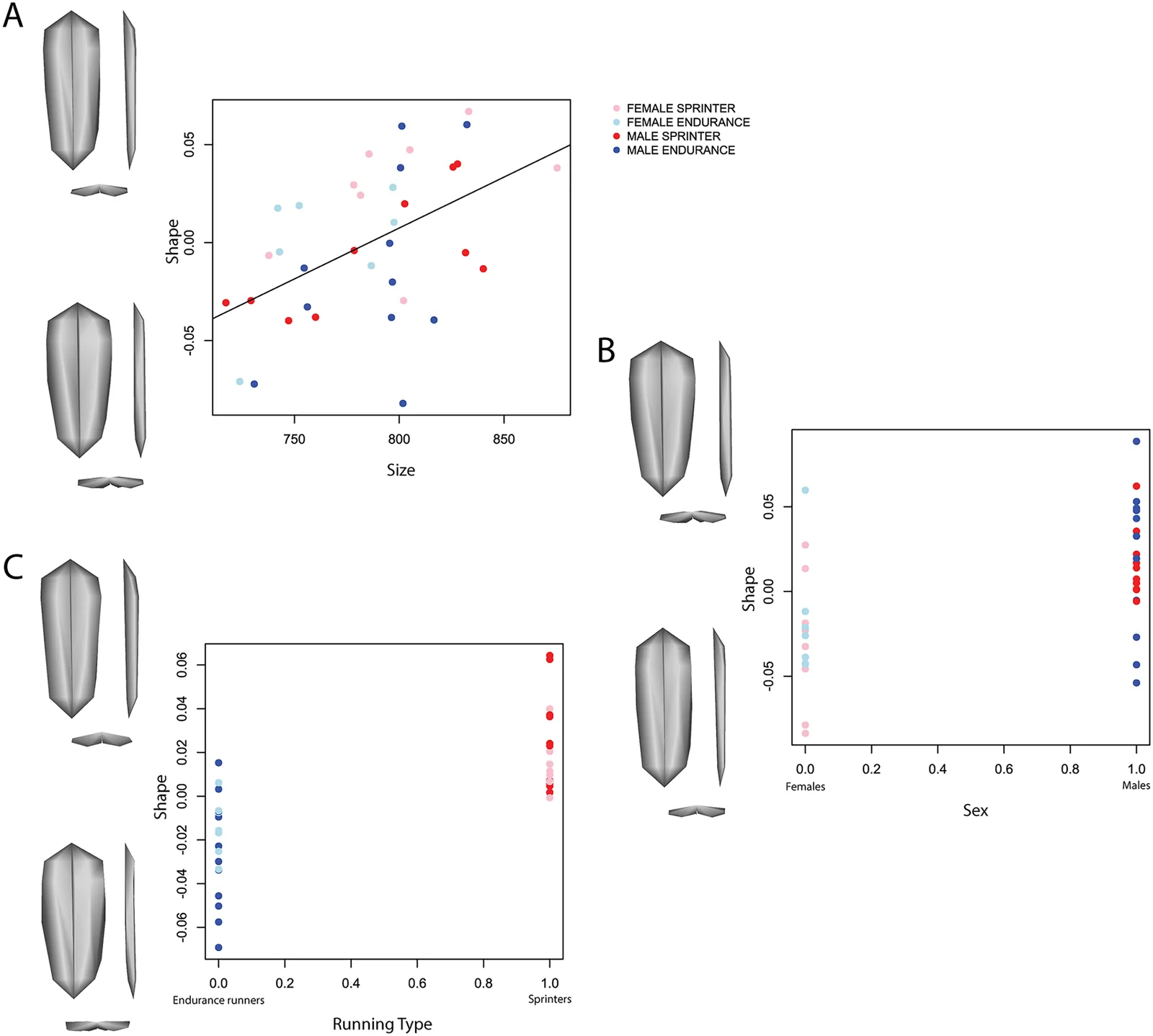
Regressions exploring the effects of size (A), sex (B) and running discipline (C), extracted from the Procrustes ANOVA model.

## Discussion

### Main findings

This preliminary study provides the first quantitative three-dimensional analysis of rectus abdominis morphology in vivo using geometric morphometrics, with an exploratory focus on morphological variation associated with allometry, sexual dimorphism and running discipline (endurance versus sprint). The PCA results show that the overall shape variation of rectus abdominis muscle can be explained by the superior shape of the muscle, its overall thickness and its convexity or concavity. The Procrustes ANOVA results confirm our main hypotheses, demonstrating that size, sex and running discipline are significantly associated with the shape variation of the rectus abdominis muscle.

Starting with shape differences related to sex (Fig 3), we can observe that females tend to show more rectangularly shaped muscles, thinner on their inferior portion, than males. Although the visual pattern suggests greater sex-related differences among sprinters, the sex × running discipline interaction was not statistically significant (S1 Table); this observation should therefore be considered descriptive and requires confirmation in larger samples.

When we explore the effect of running discipline on rectus abdominis shape, we find that endurance runners show wider, more concave muscles than sprinters. However, it is interesting to observe that males show more variance than females, in general, across a wider range of this shape change vector.

Finally, changes in shape related to centroid size, reveal that smaller muscles are wider in their superior portion and are thicker in their inferior portion. This pattern is consistent across all four groups, which exhibit a similar size range, with both large and small individuals present in every group.

These explored explanatory factors were revealed to have subtle effects. This suggests that while size, sex and running discipline significantly influence muscle shape, most of the variation is driven by other, yet unidentified factors, and these effects, although statistically significant, are modest in magnitude and should be interpreted with appropriate caution.

### Interpretations and functional considerations

The main component of shape variation of the rectus abdominis muscle was explained by the superior shape of the muscle and its convex or concave geometry. These factors are possibly related to the shape of the whole abdominal compartment, which is mainly delimited by the inferior ribcage and the pelvis. These skeletal structures of the torso show a strong covariation and morphological integration [14] and are possibly integrated with the shape of abdominal muscles such as the rectus abdominis.

The shape components extracted by the Procrustes ANOVA could potentially be related to the functionality of the rectus abdominis. In the analysis we find that there is an effect of sex on the rectus abdominis shape. This could possibly be related to known morphological constraints in females. These findings align with previous research in the shape of the skeletal torso and its integration. The shape of the rectus abdominis depends directly on the morphology of the skeletal structures at its origin (the pelvis) and insertion (the lower thorax). Males tend to exhibit wider lower thoraces than females [15], and the lower thorax is strongly integrated with the pelvic shape [16]. Females generally show wider pelves and narrower thoraces, with more declined ribs than males [15,16]. In terms of respiratory biomechanics, these characteristics in the female torso were associated with improved mechanical advantage of the ribcage which could be related to the necessity of accommodating large abdominal volume displacements, as it happens in pregnancy [17]. This constraint on the female torso and, specifically, on the pelvis is tightly related to obstetrics and could also be directly related to the role of the abdomen and its morphology. Here we find that the shape of the rectus abdominis in females is less variable than in males, which could be related to obstetric constraint and integration with the overall skeletal torso, making this one of the potential drivers of rectus abdominis dimorphic variation.

The more restricted distribution of sprinters within the PC1–PC2 morphospace raises the possibility that running discipline may be associated with specific morphological patterns. However, the cross-sectional nature of the present study does not allow us to determine whether these differences reflect training-related adaptation, pre-existing morphology, or other factors. Longitudinal studies would be required to distinguish between these possibilities. In this context, we can discuss two main functions of this muscle: the expiratory function and the core stability function. As exercise intensity increases, ventilatory demand and respiratory muscle work increase, while abdominal muscle activity may also contribute to trunk stabilization during locomotion. These combined respiratory and postural demands could therefore represent potential functional factors associated with rectus abdominis morphology. However, these mechanisms were not directly assessed in the present study.

Despite the clear importance of this muscle during extreme ventilatory and mechanical stress, its cellular profile remains largely unexplored in competitive athletes. Contrary to leg muscles, which have mainly a locomotor function and have been studied in terms of fibre-type composition in runners [18], the rectus abdominis muscle fibre composition, to our knowledge, has only been studied in normal human subjects [19,20], but not in the context of sports performance. Because abdominal muscles are less studied in sports science and exhibit greater functional complexity than leg locomotor muscles, interpretations regarding their adaptations must carefully account for this distinctive multifunctionality.

Speculatively, the morphological differences observed between endurance runners and sprinters could be associated with the different functional demands of these disciplines. It is plausible that differences in rectus abdominis shape between sprinters and endurance runners are related to demands of sprinting such as the trunk stability and strength required for maximal acceleration [21]. In fact, core stability and strength, considered collectively, have been directly related to sports performance [22].

Previous research has attempted to link muscle morphology to athletic performance. For example, Moghaddami and colleagues [23] found that while rectus abdominis was thicker in sprinters, transversus abdominis was thicker in distance runners and proposed that sprinters could use the rectus abdominis to lean during sprinting and for overall trunk stabilization. However, those findings were not corrected for body or muscle size and the results were based on a single thickness measurement at one discrete point. Regarding performance, other studies report that rectus abdominis cross sectional area does not relate to sprinting performance, at least in terms of speed [24]. This warrants caution in the functional interpretation of results.

### Potential practical applications

These findings provide a basis for future studies combining 3D muscle morphology with direct measurements of respiratory function, trunk stability and athletic performance. Such studies could determine whether the morphological differences identified here have measurable functional relevance (e.g., influencing endurance running through expiratory and pressure-generating functions, or sprint performance through trunk stability and strength).

In this study we did not use linear measurements or cross-sectional areas, because they disregard the spatial relationship of the levels measured, which are also part of the morphology of a muscle. By considering and quantifying shape we can better define the morphology of rectus abdominis and test for differences between groups.

### Strengths and limitations

This study provides the first in vivo three-dimensional geometric morphometric characterisation of the rectus abdominis muscle, revealing that size, sex and running discipline contribute to subtle variation in shape. The observed sex-related differences and the effect of running discipline highlight the importance of considering the rectus abdominis muscle within the broader morphology of the abdominal compartment and its surrounding skeletal structures, rather than relying solely on isolated linear measurements.

A major strength of this investigation lies in the recruitment of a highly specific cohort of competitive runners. However, gathering in vivo 3D data from athletic cohorts presents significant logistical challenges, and the resulting sample size of 36 competitive runners limits statistical power. The relatively small sample size and subdivision into four groups also limit the interpretation of interaction effects and the generalisation of the present findings. In addition, differences in age between groups represent a potential confounding factor that should be considered when interpreting sex- and discipline-related effects. Finally, formal intra- and inter-rater repeatability was not assessed, preventing quantitative evaluation of landmark digitization reproducibility, although landmark locations were constrained by CT slice geometry and mathematically defined cross-sectional levels.

Additionally, Procrustes ANOVA revealed a high residual variation (82.1%), indicating a substantial amount of unexplained variability. This is common in geometric morphometrics applied to biological data, where morphology is influenced by numerous factors beyond function, allometry or dimorphism [25]. In the specific case of muscle tissue, this unexplained variation likely encapsulates a myriad of unmodeled biological and lifestyle factors, such as age, nutritional status, structural asymmetries, genetic background, or individual training histories, all contributing to the large residual component.

Additionally, it should be noted that no direct measures of function were collected in this study; therefore, the functional interpretations offered here are hypotheses derived from morphology rather than confirmed physiological outcomes. Consequently, these functional interpretations should be considered with caution. Nonetheless, the Procrustes ANOVA identified significant association of size, sex and running discipline with muscle shape, providing preliminary data for future research. Future studies should expand upon these findings by including comparisons with other muscles, direct measures of performance and larger, more diverse samples to fully validate and generalise these morphological variations while improving functional interpretation.

## Conclusions

In line with the study’s main objective, our results demonstrate that the 3D shape of the rectus abdominis is significantly, though subtly, associated with size, sex and running discipline. These preliminary findings provide a basis for future research combining the study of three-dimensional muscle morphology with direct functional measurements to investigate the relationship between 3D anatomical morphology and functional outcomes in athletic performance, rehabilitation or even clinical assessment.

## Supporting information

S1 Table Procrustes ANOVA model exploring the effect of size (Centroid size), sex and running discipline on shape (with interaction between factors). (DOCX)

S2 Table STROBE checklist for observational cross-sectional studies. (DOCX)
