# Supplementary material for: The effect of size, sex and running discipline on the shape of rectus abdominis muscle: A 3D geometric morphometric approach
Source: PLoS One. 2026 Oct 28;21(10):e0358842. doi: 10.1371/journal.pone.0358842 (PMC13716172; doi:10.1371/journal.pone.0358842)
Supplement: Supplementary file 1 [file pone.0358842.s001.docx]

**S1 Table.** Procrustes ANOVA model exploring the effect of size (Centroid size), sex and running discipline on shape (with interaction between factors). Df, degrees of freedom; SS, sums of squares; MS, mean squares; R^2^, predicted variance; F, F-statistic; Z, effect size based on F distributions; p-value, significance level based on 1000 permutations. Type I (sequential) Sums of Squares were used to strictly control for allometric effects by accounting for centroid size (CS) prior to testing biological and discipline factors. Significance level*: p < 0.05.

|  | Df | SS | MS | R^2^ | F | Z | p-value |
| --- | --- | --- | --- | --- | --- | --- | --- |
| CS | 1 | 0.014 | 0.014 | 0.054 | 2.088 | 1.803 | 0.035 |
| sex | 1 | 0.016 | 0.016 | 0.062 | 2.428 | 1.989 | 0.022 |
| runningdiscipline | 1 | 0.017 | 0.017 | 0.063 | 2.450 | 2.078 | 0.015 |
| CS:sex | 1 | 0.010 | 0.010 | 0.038 | 1.491 | 1.014 | 0.160 |
| CS: runningdiscipline | 1 | 0.005 | 0.005 | 0.019 | 0.744 | -0.388 | 0.643 |
| sex: runningdiscipline | 1 | 0.007 | 0.007 | 0.028 | 1.096 | 0.447 | 0.315 |
| CS:sex: runningdiscipline | 1 | 0.004 | 0.004 | 0.015 | 0.600 | -0.942 | 0.830 |
| Residuals | 28 | 0.189 | 0.007 | 0.720 |  |  |  |
| Total | 35 | 0.262 |  |  |  |  |  |
