# Supplementary material for: The effect of size, sex and running discipline on the shape of rectus abdominis muscle: A 3D geometric morphometric approach
Source: PLoS One. 2026 Oct 28;21(10):e0358842. doi: 10.1371/journal.pone.0358842 (PMC13716172; doi:10.1371/journal.pone.0358842)
Supplement: Supplementary file 3 [file pone.0358842.s004.docx]

**Editor**

**General comments:**

This article has a small sample size; therefore, the results and conclusions should be interpreted with caution, given these important limitations.

We agree with the Editor’s assessment. However, we would like to acknowledge that while expanding the cohort is always desirable, a sample of 36 competitive athletes evaluated in vivo via 3D geometric morphometrics is rare and represents a logistically challenging dataset for this field. Nevertheless, we have revised the manuscript to address limitations of sample size, qualify the conclusions and ensure we do not over-interpret the data.

The question and methodology are interesting; however, more detail and explanation are needed. For instance, PCA suggests distinct clusters; however, substantial overlap is observed between groups. The authors should better explain the methodology used to define these clusters and clarify whether any clustering algorithm was applied or whether the PCA only represents group distributions. The ANOVA analysis demonstrated statistically significant effects of sex and running discipline on shape; however, it does not indicate that the groups form distinct clusters in morphospace. Therefore, conclusions regarding group separation should be supported by quantitative multivariate statistics. Presenting the figure for visual inspection alone is not necessary.

We appreciate this insight and the opportunity to clarify this fundamental aspect of our approach and analysis. The question the Editor raises is interesting, especially considering the methodological input of using geometric morphometric methods in this kind of translational research. We now realise more detail and explanation are needed.

First, we would like to clarify that no clustering algorithm was applied to the data, and we never intended to suggest that the groups form discrete isolated clusters in the morphospace. In geometric morphometrics, PCA is strictly used as an ordination method that helps us visualize the distribution of multivariate shape data in a reduced space, of 2 or 3 principal components that explain most of the variance of the whole data. These components usually show shared variability between groups, unless shapes are completely different. The overlap observed between groups in the PCA scatter plot is both common and biologically expected in intra-specific human soft-tissue studies, where shape variations are subtle.

For this reason, we explored the effects of the grouping variables (size, sex and running type) on shape of the muscle through a multivariate model. To this end, we used the Procrustes ANOVA as a robust quantitative multivariate statistical test. Unlike a standard univariant ANOVA, a Procrustes ANOVA evaluates the entire configuration of shape coordinates across a high-dimensional shape space. It calculates the Procrustes distances between individual specimens and group means to determine if the 3D mean shapes of the groups (e.g., males vs females) are statistically distinct. Therefore, while the groups do not form isolated clusters, the Procrustes ANOVA mathematically demonstrates that the mean shapes of these groups occupy statistically distinct positions within the complete morphospace (*p*<0.05).

Finally, regarding the PCA figure, we respectfully argue that its inclusion is necessary and standard practice in geometric morphometrics. It serves as a tool to describe the main axis of shape variation in the sample and allows the readers to visually interpret which anatomical features are being modified along the PC axes.

We have expanded the Methods and Results sections to define the multivariate nature of the Procrustes ANOVA more clearly and to clarify the descriptive and exploratory role of the PCA.

Importantly, the abstract should include a clear and explicit study objective. Although the background, methods, and main findings are described, the study's aim is not clearly stated. Based on the same comments, the study's hypothesis is also needed to clarify why the results are expected and to provide the rationale for the hypothesis (in the abstract and manuscript).

We apologize for this omission. We have revised the abstract and manuscript to explicitly state the study’s objective and hypotheses and included them in the respective sections.

**Specific comments:**

1) The functional interpretation of shape differences should be moderated, as no direct measures of respiratory function, trunk stability, core strength, or running performance were included.

We have revised the Discussion to clarify that functional interpretations are hypotheses generated from morphological analysis, not direct measurements of function. We have also softened the causal/functional language accordingly.

2) Although sex and running discipline showed statistically significant effects, the explained variance was small, and most of the shape variation remained unexplained. Please interpret with caution.

We thank the Editor for highlighting this. We have made the low effect sizes more explicit and reiterated in the Discussion that these factors, while statistically significant, explain only a small proportion of total shape variation.

3) The discussion of stronger sexual dimorphism among sprinters should be presented with caution, as the interaction term was not statistically significant.

We agree. We have revised the relevant sentences to explicitly state that the sex x discipline interaction was not statistically significant, and that the apparent stronger dimorphism in sprinters is a descriptive trend in the regression that warrants confirmation in larger samples.

4) The authors should provide more methodological detail on landmark placement, segmentation reliability, and intra- and inter-rater error.

We thank the reviewer for this important comment. We have significantly expanded the description of the landmark placement protocol in the Methods section to provide a detailed, step-by-step explanation of how the equidistant levels were calculated and how landmarks were distributed.

Regarding segmentation reliability, we would like to clarify that a standard voxel-by-voxel or pixel-by-pixel image segmentation (i.e., manual or automated tracing of the entire muscle boundary across all consecutive slices) was not performed in this study. Instead, our protocol relied entirely on a 3D geometric morphometrics approach using discrete landmark digitization at specific, mathematically defined cross-sections. Therefore, standard segmentation reliability metrics do not apply to our workflow.

In our protocol, landmark locations were not freely selected by the operator but were constrained by the CT slice geometry and mathematically defined cross-sectional levels, thereby limiting operator-dependent variability. In line with Mitteroecker (2026), repeated measurements would therefore primarily assess the repeatability of this operational measurement procedure rather than error relative to a unique “true” landmark position. Nevertheless, since landmark locations were constrained by geometry and mathematically defined cross-sectional levels on CT, rather than freely selected by the operator, we would expect operator-dependent variability to be limited. However, the absence of formal repeatability testing is acknowledged as a limitation and was added in the corresponding section of the manuscript.

5) Please include the sample size calculation.

No a priori sample size calculation was performed. This study was designed as an exploratory investigation based on the available cohort of competitive athletes with in vivo CT data. Given the novelty and logistical constraints of this 3D geometric morphometric approach, the present results should be considered preliminary and interpreted with appropriate caution. The limited sample size is now explicitly acknowledged in the Methods and Discussion.

6) For clarity and consistency with the biomedical literature, I recommend replacing the Pr(>F) column heading with P-value (or P).

Done.

7) In the first paragraph of the discussion. Please include a sentence if the results confirm the hypothesis.

Done.

8) As this is an observational study, the authors should report the manuscript in accordance with the STROBE (Strengthening the Reporting of Observational Studies in Epidemiology) guidelines.

We thank the reviewer for informing us about this requirement. This manuscript has now been reported in accordance with the STROBE guidelines (see end of methods section), and a completed STROBE checklist is provided as Supporting Information (S2 Table). Please inform us if the checklist should be modified with more information. We added page number.

9) The Discussion would benefit from the inclusion of clear subheadings, such as Potential Practical Applications, Strengths and Limitations, and Conclusions, to improve the organization and readability of the manuscript.

We thank the reviewer for this suggestion. The Discussion has been reorganised accordingly, with dedicated subheadings to improve its structure and readability.

10) In the limitations section, the authors should moderate the interpretation of the results, as this is an observational study and causal or adaptive explanations cannot be confirmed. Longitudinal studies with larger samples are needed to corroborate these findings and test whether the proposed hypotheses are supported in broader populations.

We agree with this comment. We have substantially moderated the interpretation of our findings throughout the Discussion, particularly regarding functional adaptation and causal interpretations. We now explicitly acknowledge the cross-sectional nature of the study and present the functional interpretations as hypotheses rather than demonstrated outcomes. The need for longitudinal studies with larger and more diverse samples is also emphasized.

11) What are the conclusions? The manuscript would benefit from a clear, concise conclusion that directly addresses the study's main objective.

We appreciate the suggestion to enhance the readability of the Discussion. We noticed the discussion could benefit from a better structure and order, so we rephrased the overall text to make it easier to read and follow in the sections proposed by the editor. We have carefully revised and expanded the paragraphs to ensure that the practical applications, strengths, limitations, and conclusions are clearly identifiable and follow a logical transition.

**Reviewer 1**

The manuscript is generally well structured and the methodology is clearly described. However, several issues limit interpretation, particularly regarding sample size, statistical modelling, biological interpretation, and the strength of conclusions regarding athletic adaptation and functional significance. The findings are promising but should be presented more cautiously.

We thank the Reviewer 1 for the general comment. We have reviewed the manuscript to ensure limitations are correctly addressed and conclusions are presented with caution.

The study includes 36 participants divided into four subgroups. This is a relatively small sample for testing multiple effects and interactions involving size, sex, and running discipline. Although the Procrustes ANOVA identifies significant effects of centroid size, sex, and running discipline, the explained variance is low, with residual variation accounting for the majority of shape variation (82.1%).

The groups differ substantially in age. Age is a major determinant of muscle morphology and composition. The analysis does not appear to account for age as a covariate. Without controlling for age, some observed sex or discipline effects may partially reflect demographic differences.

The study reports a significant effect of size, sex, and running discipline; however, as the R2 values indicate, these variables account for a relatively small percentage of the total shape variance. It would be beneficial to emphasize this in the results summary to ensure readers do not overstate the magnitude of these influences compared to the "unidentified factors" mentioned in the discussion. Consider supplementary allometric regressions or multivariate regression of shape on CS.

We completely agree that the sample size per subgroup is small and the high residual variation requires a more cautious interpretation of the data. In geometric morphometrics, applied to biological data, it is highly common for inter-individual variation to account for most of the variance, resulting in high residual values (Klingenberg, 2016). Muscle shape is inherently multifactorial and depends on many factors other than muscle use or sexual dimorphism, such as age, nutritional status, structural asymmetries, genetic background, etc. We have address some of these other potential explanatory factors (such as integration with skeletal structures) in the manuscript. However, incorporating all these additional variables into our statistical model with a relatively small sample size would inevitably lead to statistical overfitting and a critical depletion of the residual degrees of freedom. Despite this high residual variation, the Procrustes ANOVA successfully detected significant main effects for centroid size, sex and running discipline, although they explain only a portion of shape variation.

To fully address this point and ensure we do not imply that muscle shape is solely explained by our studied variables, we have revised the Abstract, Discussion and Conclusion sections to ensure our findings are not overstated. We also added a dedicated paragraph in the Discussion section explicitly stating the limitations of the small sample size and high residual variance, highlighting the need for future studies with larger cohorts to confirm these trends.

Authors should reduce speculative functional claims. The manuscript proposes that differences in rectus abdominis shape may relate to:sprint performance, trunk stabilization, respiratory mechanics, abdominal pressure generation, however, no direct functional measurements were collected. Therefore, statements suggesting functional specialization should be softened. For example, the conclusion that sprinter morphology may reflect adaptation to explosive acceleration is plausible but remains hypothetical.

We agree with the reviewer and since no direct functional measurements were collected, we have acknowledged in the manuscript that functional considerations must be carefully interpreted and softened the statements regarding these hypotheses.

The difference between inspiratory and expiratory configuration could itself provide important information about muscle function.

We thank the Reviewer for this excellent observation. Analysing the geometric differences between inspiration and expiration would provide valuable information regarding the dynamic and respiratory function of the muscle.

In the original manuscript we inadvertently omitted the explanation of why the TLC configuration was not utilized in our final analysis, and we are grateful to the Reviewer for bringing this to our attention. While both respiratory configurations were acquired as part of a broader project evaluating respiratory function (evaluating thorax, cervical spine, lungs and upper airways), protocols required the radiology technicians to intentionally terminate the second scan (TLC) prior to reaching the pubic symphysis to protect the gonadal region of the participants. Therefore, we restricted the rectus abdominis study to the FRC dataset.

To ensure full transparency we updated the Material and Methods section with this clarification of the protocol.

The authors state that landmark data are available as supporting information, while CT images cannot be publicly shared because of ethical restrictions.

We confirm that the landmark coordinates will be available as supporting information upon publication to ensure the reproducibility of our geometric morphometric analyses. However, the raw CT images will remain protected in strict compliance with patient privacy protocols and institutional ethical restrictions, because they contain high-resolution anatomical data from which individual participants could potentially be identified.

**References:**

Mitteroecker, P. (2026). There Is No Measurement Error in Biology. *Biological Theory*, 1-13.

Klingenberg, C. P. (2016). Size, shape, and form: concepts of allometry in geometric morphometrics. *Development genes and evolution*, *226*(3), 113-137.
