# Supplementary material for: The effect of size, sex and running discipline on the shape of rectus abdominis muscle: A 3D geometric morphometric approach
Source: PLoS One. 2026 Oct 28;21(10):e0358842. doi: 10.1371/journal.pone.0358842 (PMC13716172; doi:10.1371/journal.pone.0358842)
Supplement: Peer review history

# Peer review: Decision Letter 0

**DOI:** 10.1371/journal.pone.0358842.r001

**Authors:** Carlos Cruz-Montecinos

10 Jul 2026

Dear Dr. Gómez-Recio

Thank you for submitting your manuscript to PLOS ONE. After careful consideration, we feel that it has merit but does not fully meet PLOS ONE’s publication criteria as it currently stands. Therefore, we invite you to submit a revised version of the manuscript that addresses the points raised during the review process.

==============================

Please submit your revised manuscript by Aug 24 2026 11:59PM. If you will need more time than this to complete your revisions, please reply to this message or contact the journal office at plosone@plos.org. When you're ready to submit your revision, log on to https://www.editorialmanager.com/pone/ and select the 'Submissions Needing Revision' folder to locate your manuscript file.

- A letter that responds to each point raised by the academic editor and reviewer(s). You should upload this letter as a separate file labeled 'Response to Reviewers'.
- A marked-up copy of your manuscript that highlights changes made to the original version. You should upload this as a separate file labeled 'Revised Manuscript with Track Changes'.
- An unmarked version of your revised paper without tracked changes. You should upload this as a separate file labeled 'Manuscript'.

If you would like to make changes to your financial disclosure, please include your updated statement in your cover letter. Guidelines for resubmitting your figure files are available below the reviewer comments at the end of this letter.

If applicable, we recommend that you deposit your laboratory protocols in protocols.io to enhance the reproducibility of your results. Protocols.io assigns your protocol its own identifier (DOI) so that it can be cited independently in the future. For instructions see: https://journals.plos.org/plosone/s/submission-guidelines#loc-laboratory-protocols. Additionally, PLOS ONE offers an option for publishing peer-reviewed Lab Protocol articles, which describe protocols hosted on protocols.io. Read more information on sharing protocols at https://plos.org/protocols?utm_medium=editorial-email&utm_source=authorletters&utm_campaign=protocols.

As the corresponding author, your ORCID iD is verified in the submission system and will appear in the published article. PLOS supports the use of ORCID, and we encourage all coauthors to register for an ORCID iD and use it as well. Please encourage your coauthors to verify their ORCID iD within the submission system before final acceptance, as unverified ORCID iDs will not appear in the published article. *Only*  the individual author can complete the verification step; PLOS staff *cannot*  verify ORCID iDs on behalf of authors.

We look forward to receiving your revised manuscript.

Kind regards,

Carlos Cruz-Montecinos

Academic Editor

PLOS One

Journal requirements:

When submitting your revision, we need you to address these additional requirements.

1. Please ensure that your manuscript meets PLOS ONE's style requirements, including those for file naming. The PLOS ONE style templates can be found at

https://journals.plos.org/plosone/s/file?id=wjVg/PLOSOne_formatting_sample_main_body.pdf and

https://journals.plos.org/plosone/s/file?id=ba62/PLOSOne_formatting_sample_title_authors_affiliations.pdf

2. Thank you for stating the following financial disclosure:

“This research was supported by grant PID2020-115854GB-I00 national project to MB (funded by MCIN/AEI/10.13039/501100011033 of the Spanish Ministry of Science and Innovation) and by PIPF-2023/SAL-GL-30162 predoctoral contract to MGR (funded by the Autonomous Community of Madrid).”

Please state what role the funders took in the study.  If the funders had no role, please state: "The funders had no role in study design, data collection and analysis, decision to publish, or preparation of the manuscript."

If this statement is not correct you must amend it as needed.

Please include this amended Role of Funder statement in your cover letter; we will change the online submission form on your behalf.

3. We note that you have indicated that there are restrictions to data sharing for this study. For studies involving human research participant data or other sensitive data, we encourage authors to share de-identified or anonymized data. However, when data cannot be publicly shared for ethical reasons, we allow authors to make their data sets available upon request. For information on unacceptable data access restrictions, please see http://journals.plos.org/plosone/s/data-availability#loc-unacceptable-data-access-restrictions.

Before we proceed with your manuscript, please address the following prompts

a) If there are ethical or legal restrictions on sharing a de-identified data set, please explain them in detail (e.g., data contain potentially identifying or sensitive patient information, data are owned by a third-party organization, etc.) and who has imposed them (e.g., a Research Ethics Committee or Institutional Review Board, etc.). Please also provide contact information for a data access committee, ethics committee, or other institutional body to which data requests may be sent.

b) If there are no restrictions, please upload the minimal anonymized data set necessary to replicate your study findings to a stable, public repository and provide us with the relevant URLs, DOIs, or accession numbers. Please see http://www.bmj.com/content/340/bmj.c181.long for guidelines on how to de-identify and prepare clinical data for publication. For a list of recommended repositories, please see https://journals.plos.org/plosone/s/recommended-repositories. You also have the option of uploading the data as Supporting Information files, but we would recommend depositing data directly to a data repository if possible.

Please update your Data Availability statement in the submission form accordingly.

If the reviewer comments include a recommendation to cite specific previously published works, please review and evaluate these publications to determine whether they are relevant and should be cited. There is no requirement to cite these works unless the editor has indicated otherwise.

Additional Editor Comments:

Dear authors,

One reviewer provided valuable comments on your manuscript. I have also carefully reviewed the manuscript and have the following additional comments for your consideration.

**General comments.**

This article has a small sample size; therefore, the results and conclusions should be interpreted with caution, given these important limitations.

The question and methodology are interesting; however, more detail and explanation are needed. For instance, PCA suggests distinct clusters; however, substantial overlap is observed between groups. The authors should better explain the methodology used to define these clusters and clarify whether any clustering algorithm was applied or whether the PCA only represents group distributions. The ANOVA analysis demonstrated statistically significant effects of sex and running discipline on shape; however, it does not indicate that the groups form distinct clusters in morphospace. Therefore, conclusions regarding group separation should be supported by quantitative multivariate statistics. Presenting the figure for visual inspection alone is not necessary.

Importantly, the abstract should include a clear and explicit study objective. Although the background, methods, and main findings are described, the study's aim is not clearly stated. Based on the same comments, the study's hypothesis is also needed to clarify why the results are expected and to provide the rationale for the hypothesis (in the abstract and manuscript).

**Specific comments:**

1) The functional interpretation of shape differences should be moderated, as no direct measures of respiratory function, trunk stability, core strength, or running performance were included.

2) Although sex and running discipline showed statistically significant effects, the explained variance was small, and most of the shape variation remained unexplained. Please interpret with caution.

3) The discussion of stronger sexual dimorphism among sprinters should be presented with caution, as the interaction term was not statistically significant.

4) The authors should provide more methodological detail on landmark placement, segmentation reliability, and intra- and inter-rater error.

5) Please include the sample size calculation.

6) For clarity and consistency with the biomedical literature, I recommend replacing the Pr(>F) column heading with P-value (or P).

7) In the first paragraph of the discussion. Please include a sentence if the results confirm the hypothesis.

8) As this is an observational study, the authors should report the manuscript in accordance with the STROBE (Strengthening the Reporting of Observational Studies in Epidemiology) guidelines.

9) The Discussion would benefit from the inclusion of clear subheadings, such as Potential Practical Applications, Strengths and Limitations, and Conclusions, to improve the organization and readability of the manuscript.

10) In the limitations section, the authors should moderate the interpretation of the results, as this is an observational study and causal or adaptive explanations cannot be confirmed. Longitudinal studies with larger samples are needed to corroborate these findings and test whether the proposed hypotheses are supported in broader populations.

11) What are the conclusions? The manuscript would benefit from a clear, concise conclusion that directly addresses the study's main objective.

[Note: HTML markup is below. Please do not edit.]

Reviewers' comments:

Reviewer's Responses to Questions

**Comments to the Author**

1. Is the manuscript technically sound, and do the data support the conclusions?

Reviewer #1: Partly

**********

2. Has the statistical analysis been performed appropriately and rigorously?

Reviewer #1: Yes

**********

3. Have the authors made all data underlying the findings in their manuscript fully available?

The PLOS Data policy

Reviewer #1: No

**********

4. Is the manuscript presented in an intelligible fashion and written in standard English?

Reviewer #1: Yes

**********

Reviewer #1: The manuscript is generally well structured and the methodology is clearly described. However, several issues limit interpretation, particularly regarding sample size, statistical modelling, biological interpretation, and the strength of conclusions regarding athletic adaptation and functional significance. The findings are promising but should be presented more cautiously.

The study includes 36 participants divided into four subgroups. This is a relatively small sample for testing multiple effects and interactions involving size, sex, and running discipline. Although the Procrustes ANOVA identifies significant effects of centroid size, sex, and running discipline, the explained variance is low, with residual variation accounting for the majority of shape variation (82.1%).

The groups differ substantially in age. Age is a major determinant of muscle morphology and composition. The analysis does not appear to account for age as a covariate. Without controlling for age, some observed sex or discipline effects may partially reflect demographic differences.

The study reports a significant effect of size, sex, and running discipline; however, as the R2 values indicate, these variables account for a relatively small percentage of the total shape variance. It would be beneficial to emphasize this in the results summary to ensure readers do not overstate the magnitude of these influences compared to the "unidentified factors" mentioned in the discussion. Consider supplementary allometric regressions or multivariate regression of shape on CS.

Authors should reduce speculative functional claims. The manuscript proposes that differences in rectus abdominis shape may relate to:sprint performance, trunk stabilization, respiratory mechanics, abdominal pressure generation, however, no direct functional measurements were collected. Therefore, statements suggesting functional specialization should be softened. For example, the conclusion that sprinter morphology may reflect adaptation to explosive acceleration is plausible but remains hypothetical.

The difference between inspiratory and expiratory configuration could itself provide important information about muscle function.

The authors state that landmark data are available as supporting information, while CT images cannot be publicly shared because of ethical restrictions.

**********

what does this mean?). If published, this will include your full peer review and any attached files.

If you choose “no”, your identity will remain anonymous but your review may still be made public.

**Do you want your identity to be public for this peer review?**  For information about this choice, including consent withdrawal, please see our Privacy Policy

Reviewer #1: No

**********

[NOTE: If reviewer comments were submitted as an attachment file, they will be attached to this email and accessible via the submission site. Please log into your account, locate the manuscript record, and check for the action link "View Attachments". If this link does not appear, there are no attachment files.]

To ensure your figures meet our technical requirements, please review our figure guidelines: https://journals.plos.org/plosone/s/figures

You may also use PLOS’s free figure tool, NAAS, to help you prepare publication quality figures: https://journals.plos.org/plosone/s/figures#loc-tools-for-figure-preparation.

NAAS will assess whether your figures meet our technical requirements by comparing each figure against our figure specifications.

# Author comment: Author response to Decision Letter 1

**DOI:** 10.1371/journal.pone.0358842.r002

24 Aug 2026

Editor

General comments:

This article has a small sample size; therefore, the results and conclusions should be interpreted with caution, given these important limitations.

We agree with the Editor’s assessment. However, we would like to acknowledge that while expanding the cohort is always desirable, a sample of 36 competitive athletes evaluated in vivo via 3D geometric morphometrics is rare and represents a logistically challenging dataset for this field. Nevertheless, we have revised the manuscript to address limitations of sample size, qualify the conclusions and ensure we do not over-interpret the data.

The question and methodology are interesting; however, more detail and explanation are needed. For instance, PCA suggests distinct clusters; however, substantial overlap is observed between groups. The authors should better explain the methodology used to define these clusters and clarify whether any clustering algorithm was applied or whether the PCA only represents group distributions. The ANOVA analysis demonstrated statistically significant effects of sex and running discipline on shape; however, it does not indicate that the groups form distinct clusters in morphospace. Therefore, conclusions regarding group separation should be supported by quantitative multivariate statistics. Presenting the figure for visual inspection alone is not necessary.

We appreciate this insight and the opportunity to clarify this fundamental aspect of our approach and analysis. The question the Editor raises is interesting, especially considering the methodological input of using geometric morphometric methods in this kind of translational research. We now realise more detail and explanation are needed.

First, we would like to clarify that no clustering algorithm was applied to the data, and we never intended to suggest that the groups form discrete isolated clusters in the morphospace. In geometric morphometrics, PCA is strictly used as an ordination method that helps us visualize the distribution of multivariate shape data in a reduced space, of 2 or 3 principal components that explain most of the variance of the whole data. These components usually show shared variability between groups, unless shapes are completely different. The overlap observed between groups in the PCA scatter plot is both common and biologically expected in intra-specific human soft-tissue studies, where shape variations are subtle.

For this reason, we explored the effects of the grouping variables (size, sex and running type) on shape of the muscle through a multivariate model. To this end, we used the Procrustes ANOVA as a robust quantitative multivariate statistical test. Unlike a standard univariant ANOVA, a Procrustes ANOVA evaluates the entire configuration of shape coordinates across a high-dimensional shape space. It calculates the Procrustes distances between individual specimens and group means to determine if the 3D mean shapes of the groups (e.g., males vs females) are statistically distinct. Therefore, while the groups do not form isolated clusters, the Procrustes ANOVA mathematically demonstrates that the mean shapes of these groups occupy statistically distinct positions within the complete morphospace (p<0.05).

Finally, regarding the PCA figure, we respectfully argue that its inclusion is necessary and standard practice in geometric morphometrics. It serves as a tool to describe the main axis of shape variation in the sample and allows the readers to visually interpret which anatomical features are being modified along the PC axes.

We have expanded the Methods and Results sections to define the multivariate nature of the Procrustes ANOVA more clearly and to clarify the descriptive and exploratory role of the PCA.

Importantly, the abstract should include a clear and explicit study objective. Although the background, methods, and main findings are described, the study's aim is not clearly stated. Based on the same comments, the study's hypothesis is also needed to clarify why the results are expected and to provide the rationale for the hypothesis (in the abstract and manuscript).

We apologize for this omission. We have revised the abstract and manuscript to explicitly state the study’s objective and hypotheses and included them in the respective sections.

Specific comments:

1) The functional interpretation of shape differences should be moderated, as no direct measures of respiratory function, trunk stability, core strength, or running performance were included.

We have revised the Discussion to clarify that functional interpretations are hypotheses generated from morphological analysis, not direct measurements of function. We have also softened the causal/functional language accordingly.

2) Although sex and running discipline showed statistically significant effects, the explained variance was small, and most of the shape variation remained unexplained. Please interpret with caution.

We thank the Editor for highlighting this. We have made the low effect sizes more explicit and reiterated in the Discussion that these factors, while statistically significant, explain only a small proportion of total shape variation.

3) The discussion of stronger sexual dimorphism among sprinters should be presented with caution, as the interaction term was not statistically significant.

We agree. We have revised the relevant sentences to explicitly state that the sex x discipline interaction was not statistically significant, and that the apparent stronger dimorphism in sprinters is a descriptive trend in the regression that warrants confirmation in larger samples.

4) The authors should provide more methodological detail on landmark placement, segmentation reliability, and intra- and inter-rater error.

We thank the reviewer for this important comment. We have significantly expanded the description of the landmark placement protocol in the Methods section to provide a detailed, step-by-step explanation of how the equidistant levels were calculated and how landmarks were distributed.

Regarding segmentation reliability, we would like to clarify that a standard voxel-by-voxel or pixel-by-pixel image segmentation (i.e., manual or automated tracing of the entire muscle boundary across all consecutive slices) was not performed in this study. Instead, our protocol relied entirely on a 3D geometric morphometrics approach using discrete landmark digitization at specific, mathematically defined cross-sections. Therefore, standard segmentation reliability metrics do not apply to our workflow.

In our protocol, landmark locations were not freely selected by the operator but were constrained by the CT slice geometry and mathematically defined cross-sectional levels, thereby limiting operator-dependent variability. In line with Mitteroecker (2026), repeated measurements would therefore primarily assess the repeatability of this operational measurement procedure rather than error relative to a unique “true” landmark position. Nevertheless, since landmark locations were constrained by geometry and mathematically defined cross-sectional levels on CT, rather than freely selected by the operator, we would expect operator-dependent variability to be limited. However, the absence of formal repeatability testing is acknowledged as a limitation and was added in the corresponding section of the manuscript.

5) Please include the sample size calculation.

No a priori sample size calculation was performed. This study was designed as an exploratory investigation based on the available cohort of competitive athletes with in vivo CT data. Given the novelty and logistical constraints of this 3D geometric morphometric approach, the present results should be considered preliminary and interpreted with appropriate caution. The limited sample size is now explicitly acknowledged in the Methods and Discussion.

6) For clarity and consistency with the biomedical literature, I recommend replacing the Pr(>F) column heading with P-value (or P).

Done.

7) In the first paragraph of the discussion. Please include a sentence if the results confirm the hypothesis.

Done.

8) As this is an observational study, the authors should report the manuscript in accordance with the STROBE (Strengthening the Reporting of Observational Studies in Epidemiology) guidelines.

We thank the reviewer for informing us about this requirement. This manuscript has now been reported in accordance with the STROBE guidelines (see end of methods section), and a completed STROBE checklist is provided as Supporting Information (S2 Table). Please inform us if the checklist should be modified with more information. We added page number.

9) The Discussion would benefit from the inclusion of clear subheadings, such as Potential Practical Applications, Strengths and Limitations, and Conclusions, to improve the organization and readability of the manuscript.

We thank the reviewer for this suggestion. The Discussion has been reorganised accordingly, with dedicated subheadings to improve its structure and readability.

10) In the limitations section, the authors should moderate the interpretation of the results, as this is an observational study and causal or adaptive explanations cannot be confirmed. Longitudinal studies with larger samples are needed to corroborate these findings and test whether the proposed hypotheses are supported in broader populations.

We agree with this comment. We have substantially moderated the interpretation of our findings throughout the Discussion, particularly regarding functional adaptation and causal interpretations. We now explicitly acknowledge the cross-sectional nature of the study and present the functional interpretations as hypotheses rather than demonstrated outcomes. The need for longitudinal studies with larger and more diverse samples is also emphasized.

11) What are the conclusions? The manuscript would benefit from a clear, concise conclusion that directly addresses the study's main objective.

We appreciate the suggestion to enhance the readability of the Discussion. We noticed the discussion could benefit from a better structure and order, so we rephrased the overall text to make it easier to read and follow in the sections proposed by the editor. We have carefully revised and expanded the paragraphs to ensure that the practical applications, strengths, limitations, and conclusions are clearly identifiable and follow a logical transition.

Reviewer 1

The manuscript is generally well structured and the methodology is clearly described. However, several issues limit interpretation, particularly regarding sample size, statistical modelling, biological interpretation, and the strength of conclusions regarding athletic adaptation and functional significance. The findings are promising but should be presented more cautiously.

We thank the Reviewer 1 for the general comment. We have reviewed the manuscript to ensure limitations are correctly addressed and conclusions are presented with caution.

The study includes 36 participants divided into four subgroups. This is a relatively small sample for testing multiple effects and interactions involving size, sex, and running discipline. Although the Procrustes ANOVA identifies significant effects of centroid size, sex, and running discipline, the explained variance is low, with residual variation accounting for the majority of shape variation (82.1%).

The groups differ substantially in age. Age is a major determinant of muscle morphology and composition. The analysis does not appear to account for age as a covariate. Without controlling for age, some observed sex or discipline effects may partially reflect demographic differences.

The study reports a significant effect of size, sex, and running discipline; however, as the R2 values indicate, these variables account for a relatively small percentage of the total shape variance. It would be beneficial to emphasize this in the results summary to ensure readers do not overstate the magnitude of these influences compared to the "unidentified factors" mentioned in the discussion. Consider supplementary allometric regressions or multivariate regression of shape on CS.

We completely agree that the sample size per subgroup is small and the high residual variation requires a more cautious interpretation of the data. In geometric morphometrics, applied to biological data, it is highly common for inter-individual variation to account for most of the variance, resulting in high residual values (Klingenberg, 2016). Muscle shape is inherently multifactorial and depends on many factors other than muscle use or sexual dimorphism, such as age, nutritional status, structural asymmetries, genetic background, etc. We have address some of these other potential explanatory factors (such as integration with skeletal structures) in the manuscript. However, incorporating all these additional variables into our statistical model with a relatively small sample size would inevitably lead to statistical overfitting and a critical depletion of the residual degrees of freedom. Despite this high residual variation, the Procrustes ANOVA successfully detected significant main effects for centroid size, sex and running discipline, although they explain only a portion of shape variation.

To fully address this point and ensure we do not imply that muscle shape is solely explained by our studied variables, we have revised the Abstract, Discussion and Conclusion sections to ensure our findings are not overstated. We also added a dedicated paragraph in the Discussion section explicitly stating the limitations of the small sample size and high residual variance, highlighting the need for future studies with larger cohorts to confirm these trends.

Authors should reduce speculative functional claims. The manuscript proposes that differences in rectus abdominis shape may relate to:sprint performance, trunk stabilization, respiratory mechanics, abdominal pressure generation, however, no direct functional measurements were collected. Therefore, statements suggesting functional specialization should be softened. For example, the conclusion that sprinter morphology may reflect adaptation to explosive acceleration is plausible but remains hypothetical.

We agree with the reviewer and since no direct functional measurements were collected, we have acknowledged in the manuscript that functional considerations must be carefully interpreted and softened the statements regarding these hypotheses.

The difference between inspiratory and expiratory configuration could itself provide important information about muscle function.

We thank the Reviewer for this excellent observation. Analysing the geometric differences between inspiration and expiration would provide valuable information regarding the dynamic and respiratory function of the muscle.

In the original manuscript we inadvertently omitted the explanation of why the TLC configuration was not utilized in our final analysis, and we are grateful to the Reviewer for bringing this to our attention. While both respiratory configurations were acquired as part of a broader project evaluating respiratory function (evaluating thorax, cervical spine, lungs and upper airways), protocols required the radiology technicians to intentionally terminate the second scan (TLC) prior to reaching the pubic symphysis to protect the gonadal region of the participants. Therefore, we restricted the rectus abdominis study to the FRC dataset.

To ensure full transparency we updated the Material and Methods section with this clarification of the protocol.

The authors state that landmark data are available as supporting information, while CT images cannot be publicly shared because of ethical restrictions.

We confirm that the landmark coordinates will be available as supporting information upon publication to ensure the reproducibility of our geometric morphometric analyses. However, the raw CT images will remain protected in strict compliance with patient privacy protocols and institutional ethical restrictions, because they contain high-resolution anatomical data from which individual participants could potentially be identified.

References:

Mitteroecker, P. (2026). There Is No Measurement Error in Biology. Biological Theory, 1-13.

Klingenberg, C. P. (2016). Size, shape, and form: concepts of allometry in geometric morphometrics. Development genes and evolution, 226(3), 113-137.

Attachment Submitted filename: Response to Reviewers.docx

# Peer review: Decision Letter 1

**DOI:** 10.1371/journal.pone.0358842.r003

**Authors:** Marko Joksimovic

7 Sep 2026

The effect of size, sex and running discipline on the shape of rectus abdominis muscle: a 3D geometric morphometric approach

PONE-D-26-12271R1

Dear Dr. Gómez-Recio,

We’re pleased to inform you that your manuscript has been judged scientifically suitable for publication and will be formally accepted for publication once it meets all outstanding technical requirements.

Within one week, you’ll receive an e-mail detailing the required amendments. When these have been addressed, you’ll receive a formal acceptance letter and your manuscript will be scheduled for publication.

An invoice will be generated when your article is formally accepted. Please note, if your institution has a publishing partnership with PLOS and your article meets the relevant criteria, all or part of your publication costs will be covered. Please make sure your user information is up-to-date by logging into Editorial Manager at Editorial Manager® and clicking the ‘Update My Information' link at the top of the page. For questions related to billing, please contact billing support.

If your institution or institutions have a press office, please notify them about your upcoming paper to help maximize its impact. If they’ll be preparing press materials, please inform our press team as soon as possible -- no later than 48 hours after receiving the formal acceptance. Your manuscript will remain under strict press embargo until 2 pm Eastern Time on the date of publication. For more information, please contact onepress@plos.org.

Kind regards,

Marko Joksimovic

Academic Editor

PLOS One

Additional Editor Comments (optional):

I confirm that the authors have thoroughly and adequately addressed all comments and suggestions from the reviewers and have incorporated all requested corrections into the manuscript. The paper has been significantly improved and fully meets the scientific and methodological standards of the journal. Given the above, I recommend that the paper be accepted for publication.

Reviewers' comments:

Reviewer's Responses to Questions

**Comments to the Author**

Reviewer #1: All comments have been addressed

Reviewer #2: (No Response)

**********

2. Is the manuscript technically sound, and do the data support the conclusions?

Reviewer #1: Yes

Reviewer #2: Partly

**********

3. Has the statistical analysis been performed appropriately and rigorously?

Reviewer #1: Yes

Reviewer #2: No

**********

4. Have the authors made all data underlying the findings in their manuscript fully available?

The PLOS Data policy

Reviewer #1: Yes

Reviewer #2: (No Response)

**********

5. Is the manuscript presented in an intelligible fashion and written in standard English?

Reviewer #1: Yes

Reviewer #2: (No Response)

**********

Reviewer #1: The authors have addressed the core scientific and reporting concerns raised previously. Once the residual linguistic caution is applied in the Abstract and a couple of clarifying sentences are added regarding age and residual variance, the manuscript will be suitable for publication in PLOS ONE. The work provides a valuable methodological and descriptive foundation for future studies that combine 3D muscle morphology with direct functional measurements.

Reviewer #2: The manuscript addresses an interesting question using a novel 3D geometric morphometric approach. The overall concept is strong, but several aspects should be clarified or revised.

- Please state clearly what was hypothesized regarding each factor.

- There are substantial differences in mean age between groups. If the raw data permit, please consider including age as a covariate or conducting a sensitivity analysis. If this is not possible, this issue should be explicitly acknowledged as a limitation.

- Although centroid size, sex, and running discipline are statistically significant, they explain a small percentage of shape variation, respectively. Please emphasize the relatively small magnitude of these effects and avoid implying that these factors account for a large proportion of morphological variation.

- The manuscript states that sexual dimorphism appears stronger among sprinters, while also acknowledging that the interaction was not statistically significant. A nonsignificant interaction does not establish that the magnitude of the sex effect differs between disciplines. Please present this observation as exploratory rather than as evidence of a confirmed interaction.

- Please provide clearer definitions of the landmarks, distinguish anatomical from geometric landmarks, describe the digitization procedure, and report intra-/inter-observer error if these assessments were performed. A measurement-error analysis would considerably strengthen the study.

- The Methods indicate that the seven levels divide the rectus abdominis into right and left portions. Please explain whether both sides were included in the same configuration, whether bilateral asymmetry was assessed, and how correspondence between left and right landmarks was established.

- Please provide objective criteria for group assignment, including the running distances/events considered "sprinting" and "endurance."

- The study does not directly measure respiratory muscle activation, expiratory pressure, trunk stability, VO₂max, sprint speed, or athletic performance. Therefore, proposed relationships between rectus abdominis shape and respiratory or performance functions should be presented as hypotheses rather than demonstrated effects.

- Statements such as the suggestion of "functional adaptation" are difficult to support with a cross-sectional morphological study. Please use more cautious language.

- The proposed relationship between female rectus abdominis morphology and obstetric constraints is interesting, but pelvic morphology, pregnancy history, and obstetric variables were not directly measured. This explanation should therefore be clearly identified as a hypothesis requiring future testing.

- Please add a dedicated limitations paragraph covering sample size, subgroup imbalance, age differences, cross-sectional design, absence of direct functional/performance measures, training history, landmark measurement error, and other potentially relevant unmeasured factors.

- Improved title: Association of size, sex, and running discipline with rectus abdominis muscle shape: a three-dimensional geometric morphometric study

**********

what does this mean?). If published, this will include your full peer review and any attached files.

If you choose “no”, your identity will remain anonymous but your review may still be made public.

**Do you want your identity to be public for this peer review?**  For information about this choice, including consent withdrawal, please see our Privacy Policy

Reviewer #1: No

Reviewer #2: No

**********

# Editor report: Acceptance letter

**DOI:** 10.1371/journal.pone.0358842.r004

**Authors:** Marko Joksimovic

PONE-D-26-12271R1

PLOS One

Dear Dr. Gómez-Recio,

I'm pleased to inform you that your manuscript has been deemed suitable for publication in PLOS One. Congratulations! Your manuscript is now being handed over to our production team.

At this stage, our production department will prepare your paper for publication. This includes ensuring the following:

* All references, tables, and figures are properly cited

* All relevant supporting information is included in the manuscript submission,

* There are no issues that prevent the paper from being properly typeset

You will receive further instructions from the production team, including instructions on how to review your proof when it is ready. Please keep in mind that we are working through a large volume of accepted articles, so please give us a few days to review your paper and let you know the next and final steps.

Lastly, if your institution or institutions have a press office, please let them know about your upcoming paper now to help maximize its impact. If they'll be preparing press materials, please inform our press team within the next 48 hours. Your manuscript will remain under strict press embargo until 2 pm Eastern Time on the date of publication. For more information, please contact onepress@plos.org.

You will receive an invoice from PLOS for your publication fee after your manuscript has reached the completed accept phase. If you receive an email requesting payment before acceptance or for any other service, this may be a phishing scheme. Learn how to identify phishing emails and protect your accounts at https://explore.plos.org/phishing.

If we can help with anything else, please email us at customercare@plos.org.

Thank you for submitting your work to PLOS One and supporting open access.

Kind regards,

PLOS One Editorial Office Staff

on behalf of

Dr. Marko Joksimovic

Academic Editor

PLOS One
